# Volume Tracking: A new method for quantitative assessment and visualization of intracardiac blood flow from three-dimensional, time-resolved, three-component magnetic resonance velocity mapping

**DOI:** 10.1186/1471-2342-11-10

**Published:** 2011-04-12

**Authors:** Johannes Töger, Marcus Carlsson, Gustaf Söderlind, Håkan Arheden, Einar Heiberg

**Affiliations:** 1 Department of Clinical Physiology, Lund University, Skåne University Hospital Lund, Lund, Sweden; 2 Department of Numerical Analysis, Centre for Mathematical Sciences, Lund University, Lund, Sweden

## Abstract

**Background:**

Functional and morphological changes of the heart influence blood flow patterns. Therefore, flow patterns may carry diagnostic and prognostic information. Three-dimensional, time-resolved, three-directional phase contrast cardiovascular magnetic resonance (4D PC-CMR) can image flow patterns with unique detail, and using new flow visualization methods may lead to new insights. The aim of this study is to present and validate a novel visualization method with a quantitative potential for blood flow from 4D PC-CMR, called Volume Tracking, and investigate if Volume Tracking complements particle tracing, the most common visualization method used today.

**Methods:**

Eight healthy volunteers and one patient with a large apical left ventricular aneurysm underwent 4D PC-CMR flow imaging of the whole heart. Volume Tracking and particle tracing visualizations were compared visually side-by-side in a visualization software package. To validate Volume Tracking, the number of particle traces that agreed with the Volume Tracking visualizations was counted and expressed as a percentage of total released particles in mid-diastole and end-diastole respectively. Two independent observers described blood flow patterns in the left ventricle using Volume Tracking visualizations.

**Results:**

Volume Tracking was feasible in all eight healthy volunteers and in the patient. Visually, Volume Tracking and particle tracing are complementary methods, showing different aspects of the flow. When validated against particle tracing, on average 90.5% and 87.8% of the particles agreed with the Volume Tracking surface in mid-diastole and end-diastole respectively. Inflow patterns in the left ventricle varied between the subjects, with excellent agreement between observers. The left ventricular inflow pattern in the patient differed from the healthy subjects.

**Conclusion:**

Volume Tracking is a new visualization method for blood flow measured by 4D PC-CMR. Volume Tracking complements and provides incremental information compared to particle tracing that may lead to a better understanding of blood flow and may improve diagnosis and prognosis of cardiovascular diseases.

## Background

Blood flow patterns are closely linked to the function of valves, vessels and the myocardium, and functional and morphological changes in the heart influence the flow patterns [1-4]. Thus a better understanding of flow patterns may lead to improved diagnosis and prognosis of cardiovascular diseases.

Cardiovascular Magnetic Resonance (CMR) enables the measurement of the full three-dimensional and time-resolved velocity field in the heart and great vessels using four-dimensional Phase Contrast Cardiovascular Magnetic Resonance (4D PC-CMR) [5-7]. Due to the complexity of 4D flow fields, there exists no single natural representation that can show all details of the flow. To handle this abundance of information, flow visualization is often used for discovery, understanding and communication about flow [8]. Visualization is a critical part of the discovery process, as it enables the researcher to interact with the complex data, and by using the powerful pattern recognition systems of the human visual system, new hypotheses about the flow can be formed and then be investigated scientifically. In the case of three-dimensional, time-resolved (4D) flow, visualization is not only beneficial, but a necessity, since it would be an extremely time-consuming and error-prone task to interpret the flow using two-dimensional slices of the velocity volume [9].

Several flow visualization methods have been developed, each showing different aspects of the flow [10,11]. The most commonly used method for visualization of 4D PC-CMR data is particle tracing [9,12], but streamlines, vector plots [13] and vortex cores [14] have also been used. Methods for quantitative analysis have also been presented, including retrospective flow quantification [15], relative pressure mapping [16], turbulence mapping [6], wall shear stress [17], separation of left ventricular flow into components [18] and quantification of helical flow [19].

Visualization plays an important role in generating hypotheses that guide research. The result and performance of flow visualizations has been shown to be heavily influenced by the choice of flow visualization method [20] and even by minor adjustments in the visualization methods themselves [21]. This means that the impression of the flow changes with the visualization method, and therefore also the hypotheses generated and subsequently the studies performed and conclusions drawn. Since a single method may not show all aspects of the flow, research in alternative methods for blood flow visualization is motivated.

We have therefore developed a new visualization method, called Volume Tracking. Volume Tracking is similar to particle tracing in that it displays the path taken by the blood through the circulation, but follows and displays volumes and surfaces instead of points, changing the visual representation of the flow. Additionally, Volume Tracking adds a quantitative component to the visualizations. The purposes of this article are *1*. To present and validate a newly developed flow visualization method, Volume Tracking, and *2*. To investigate if Volume Tracking complements particle tracing and provides incremental information.

## Methods

### Study population and CMR protocol

Eight healthy volunteers were included in the study. All volunteers had normal blood pressure, normal ECG and no history of cardiovascular disease. One patient was also included to investigate if Volume Tracking was feasible in patients. This patient was hypertensive and had an apical aneurysm that developed after a major anterior infarction, resulting in an LV ejection fraction of 15%. A standard CMR examination consisting of Cine images in the 2-chamber, 3-chamber, 4-chamber and short-axis views was performed on all subjects. An experienced observer verified normal anatomy, myocardial function and valve function in the healthy volunteers. The study was approved by the Regional Ethical Review Board in Lund, Sweden. Written informed consent was obtained from all subjects. Subject characteristics are summarized in Table 1.

**Table 1:** Subject characteristics.

	Subject	Age (years)	Gender	Weight (kg)	Height (cm)
Normals:	1	52	F	57	160

	2	23	M	89	194

	3	28	M	64	173

	4	36	F	70	177

	5	29	M	88	185

	6	63	M	70	170

	7	24	M	85	188

	8	25	F	67	165

	*n *= 8	23 - 63	5M, 3F	57 - 89	160 - 194

Patient:	AA	74	M	83	180

Characteristics for the subjects included in the study. AA = Apical Aneurysm.

Three-dimensional, time-resolved, three-component velocity mapping (4D PC-CMR) was performed over the whole heart on all subjects using a three-dimensional Turbo Field Echo (TFE) Phase Contrast sequence [6] on a Philips Achieva 3T CMR scanner. The 4D flow and cine images were acquired in the same imaging session, so that the cine images could be used as spatial orientation when visualizing the 4D flow data. For the 4D flow measurements, the following parameters were used: Spatial resolution 3 × 3 × 3 mm, flip angle 8°, TE 3.7 ms, TR 6.2 ms, velocity encoding (VENC) 100 cm/s, and temporal resolution typically 50 ms. Parallel imaging (SENSE) was used with a factor of 2, and the turbo factor was 2. Respiratory gating to the end-expiratory phase was performed with a navigator beam for the diaphragm. Synchronization to the cardiac cycle was performed using retrospective ECG triggering, covering the whole R-R interval. All imaging was performed with the subjects at rest. Scan time for the flow measurement was typically 35 minutes. The flow measurement and standard CMR examination were performed in the same session, bringing the total scan time to about 50 minutes.

### Volume Tracking

This section presents an overview of the Volume Tracking theory and implementation. The full mathematical formulation and technical details of Volume Tracking, necessary to reproduce and analyze the method, are given in Additional File 1: VT-Appendix.pdf.

Volume Tracking is based on the concept of a *flow map *, which is a function that maps the location  of a particle at one time *t*_0 _to its position at another time *t*_1_, thereby answering the question "Where will this particle go?" or "Where did this particle come from?". Using the flow map, the evolution of any volume in the flow can be defined: If the volume at the starting time *t*_0 _is *V*_0_, and the volume at another time *t *is *V*(*t*), we define *V*(*t*) as the image of *V*_0 _under the flow map as

To compute the flow map, an auxiliary function  is introduced, mapping particles from their current position to their positions at time *t*_0_, the *starting time*. Denoting the velocity field by , and decomposing *ψ *into its cartesian components (*ψ^x^*, *ψ^y^*, *ψ^z^*), and decomposing  = (*x*, *y*, *z*), the advection equations(1)

then describe the motion of volumes in the flow. The initial conditions at *t *= *t*_0 _are

These equations can be efficiently solved using a finite volume method, further described Additional File 1: VT-Appendix.pdf.

A starting volume *V*_0 _is specified using a *volume function f*:

and the evolution of a volume can then be computed as(2)

The solution to the Volume Tracking equations can be pre-computed, and the evolution of any volume is encoded in the flow map. Blood volumes of different shape, such as spheres and planes, can then be followed forward or backward in time interactively. Using the current implementation of Volume Tracking, the pre-computation step typically lasts 10-15 minutes per starting time. However, the pre-computation step can be performed offline without human interaction once the starting times have been chosen. Once the pre-computation has been completed, the flow map can be used to select volumes of several shapes and any position, and the movement and deformation of this volume can be displayed instantaneously.

The full mathematical formulation and technical details of Volume Tracking, necessary to reproduce and analyze the method, are given in Additional File 1: VT-Appendix.pdf.

Volume Tracking was implemented in MATLAB (The Mathworks, USA), using the freely available Finite Volume solver CLAWPACK [22-24] for the numerical calculations. User interaction, image display and animation were performed in the visualization software package Ensight (CEI, USA).

### Software

To correct for eddy currents and other phase background effects, a first-order polynomial was fitted to velocities in stationary tissue and subtracted from the acquired velocity field [25]. Additionally, automatic phase unwrapping was performed to correct for phase aliasing effects [26]. Concomitant gradient effects were compensated by the CMR scanner software. Correction of background effects and phase unwrapping of the velocity data was implemented as a plugin to Segment [27], a freely available software for cardiovascular image analysis based on MATLAB (The Mathworks, USA). The visualization software package Ensight 9.1 (CEI, USA) was used to display Volume Tracking surfaces, compute particle traces and to create images and animations. Particle traces were computed using Ensight's default fourth-order Runge-Kutta integration algorithm [28], which uses linear interpolation between timesteps and grid points. The implementation of Volume Tracking is described in Additional File 1: VT-Appendix.pdf.

### Evaluation and validation of Volume Tracking

Particle tracing and Volume Tracking both display the path taken by blood through the cardiovascular system, although giving different visual impressions of the flow. Therefore, to evaluate the visualization aspects of Volume Tracking, Volume Tracking and particle trace visualizations were generated in the visualization software package Ensight (CEI, USA) and compared side-by-side. Side-by-side visualizations were generated for diastolic inflow in left ventricle and right ventricle in a healthy volunteer, and diastolic inflow in the left ventricle in the ischemic cardiomyopathy patient. In the visualizations of left ventricular flow, the Volume Tracking surface shows the boundary between inflowing blood and blood already in the ventricle from the previous heartbeat.

**Figure 1:**
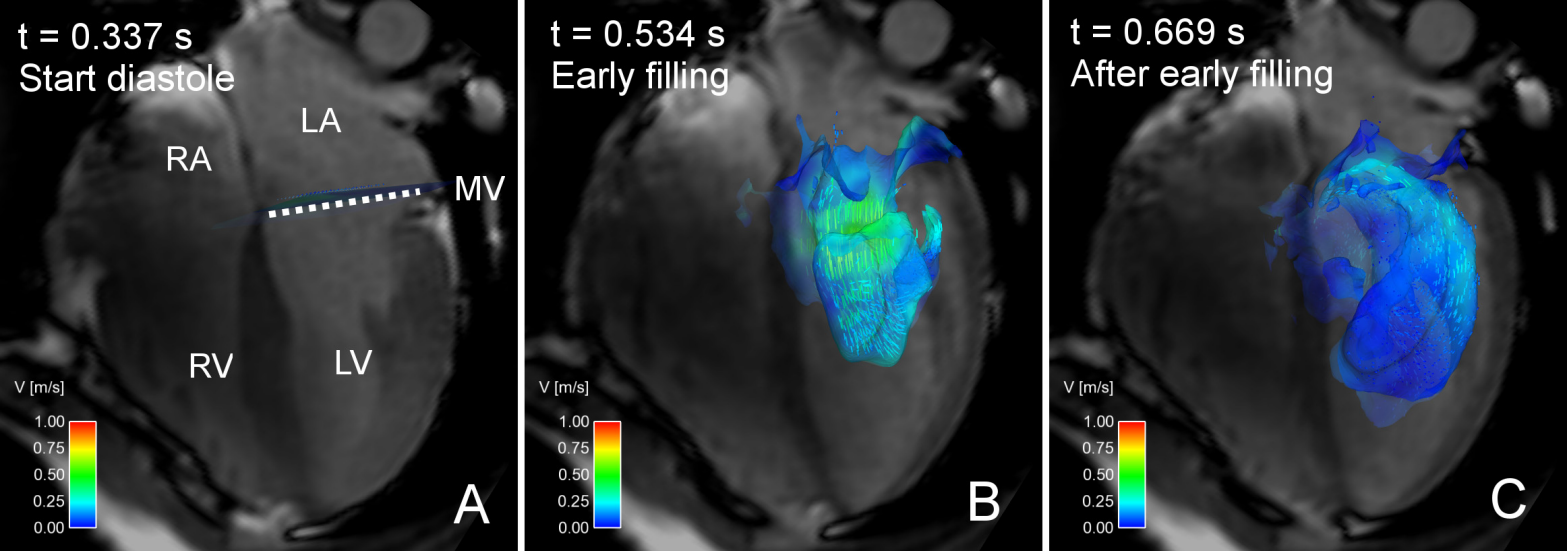
**Composite visualization using both Volume Tracking and particle tracing**. Combined particle tracing and Volume Tracking visualization of LV filling in volunteer 7. See Additional File 2: VT-PT-Combination.mpg for an animated version. In each image, an anatomical 4-chamber Cine image is shown for orientation. The 4-chamber image is transparent to show flow behind the plane. Time is counted from the start of systole. Panel A shows the heart at the start of diastole. A Volume Tracking plane is visible near the mitral valve, and a collection of particles is located above the plane. Particles are released in this position every 20 milliseconds during LV filling. Panel B shows the early filling wave. The Volume Tracking surface shows the inflowing blood volume. Particles have been released in the atrium and have flowed into the ventricle. The particles are visible through the transparent Volume Tracking surface. Panel C shows the flow in early diastasis. The Volume Tracking surface shows how the blood has flowed further into the ventricle. The particles have also moved further into the ventricle. Note that the particles are released on the basal side of the Volume Tracking plane. This means that all particles belong to the blood flowing into the ventricle. Since the Volume Tracking surface shows the inflowing blood, the particles should stay on the inflow side of the surface if the methods agree. Panels B and C show that very few particles have passed through the surface. This shows the correspondence between Volume Tracking and particle tracing, and that the theory and implementation of Volume Tracking are reliable. *LV = left ventricle, RV = right ventricle, LA = left atrium, RA = right atrium, MV = mitral valve, dashed line = approximate location of mitral valve, color = velocity from 0 (blue) to 1 m/s (red)*.

To validate Volume Tracking against particle tracing, combined visualizations were generated of LV inflow in all subjects, with Volume Tracking and particle tracing displayed superimposed in the same image (see Figure 1 and Additional File 2: VT-PT-Combination.mpg). Since both Volume Tracking and particle tracing compute and display the motion of blood through the heart, no particles should cross the Volume Tracking surface if the methods display the exact same blood motion. Therefore, a particle was defined as fulfilling the validation criterion if it was located on the same side of the Volume Tracking plane as it was released on. The agreement between Volume Tracking and particle tracing at mid-diastole and end-diastole was calculated as the number of particles fulfilling the validation criterion, divided by the number of particles released up to that timepoint, and expressed as a percentage. At least 1000 particles were released in each subject. To ensure that this validation methodology was not sensitive to the number of released particles, the number of particles was varied between 1000 and 8000 in subject 7.

### Analysis of LV inflow patterns

**Figure 2:**
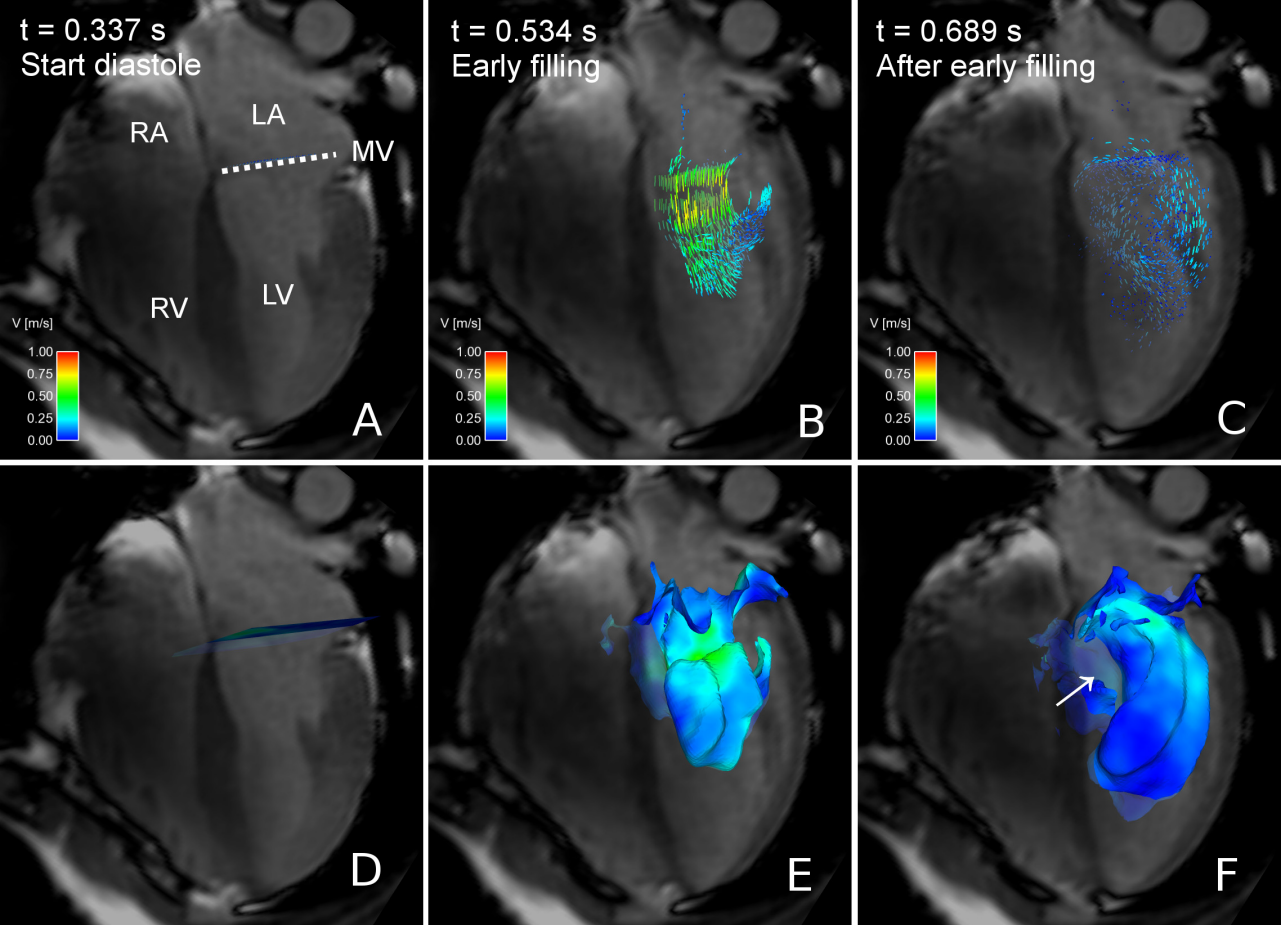
**Particle tracing and Volume Tracking visualizations of LV filling**. Particle tracing (A-C) and Volume Tracking (D-F) visualizations of LV filling in volunteer 7. See Additional File 3: VT-Comparison.mpg for an animated version. In each image, an anatomical 4-chamber Cine image is shown for orientation. The 4-chamber image is transparent to show flow behind the plane. Time is counted from the start of ventricular systole. Panels A-C show the particle tracing visualization. Particles are released every 20 milliseconds at the level of the white dotted line (MV). In panel A, at the start of diastole, particles can be seen at their starting position just above the dotted white line. In panel B, during the early phase of the filling, the particle traces flow from the atrium into the ventricle. In panel C, during diastasis, the particles have decelerated and dispersed in the ventricle. Panels D-F show the Volume Tracking visualization corresponding to panels A-C. Panel D shows a plane at the level of the mitral valve just before ventricular diastole. In panel E, Volume Tracking shows the volume of blood flowing into the ventricle. In panel F, the blood has flowed further into the ventricle. Panels E and F show how the Volume Tracking plane is deformed as if it were an in finitely stretchable, flexible sheet, i.e. the Volume Tracking visualization shows the boundary between blood flowing into the ventricle and blood already in the ventricle. Panel F clearly shows an absence of filling blood in an area near the basal parts of the septum (white arrow), something which is not easily seen in the corresponding particle trace image (panel C). *LV = left ventricle, RV = right ventricle, LA = left atrium, RA = right atrium, MV = mitral valve, dotted line = approximate location of mitral valve, color = velocity from 0 (blue) to 1 m/s (red)*.

**Figure 3:**
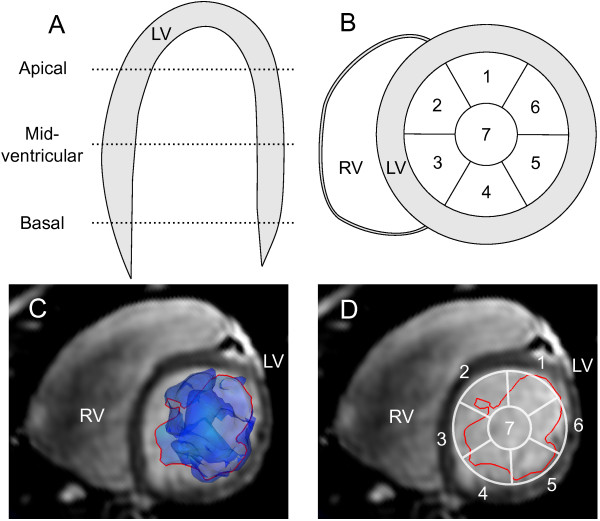
**Division of the LV lumen into segments**. In panel A the LV lumen is divided into apical, mid-ventricular and basal parts along the long-axis. Panel B shows how each short-axis part is divided into seven segments, where six (labels 1-6) are located along the endocardial border adjusted from the AHA standard [29], and the seventh (label 7) in the center of the lumen. Panel C shows a representative slice and Volume Tracking surface (blue transparent surface). The intersection of the Volume Tracking surface with the short-axis slice is shown as a red line. Panel D shows the intersection in red and the segment model from panel B in white. *LV = left ventricle, RV = right ventricle, 1-7 = segment numbers*.

To demonstrate one of the possible applications of Volume Tracking, Volume Tracking visualizations were used to describe blood flow patterns in the left ventricle (LV). Using a Volume Tracking plane, as shown in Figure 2, panels D-E, the boundary between blood flowing into the ventricle and blood already in the ventricle was determined for the whole diastolic period. To map the location of the inflow blood, the LV lumen was divided into segments according to Figure 3. The LV lumen was divided into Apical, Mid-ventricular and Basal parts along the long-axis (Figure 3, panel A). Each part was then divided into seven segments (Figure 3, panel B) adjusted from the AHA model [29]. The intersection between the Volume Tracking surface and short-axis slices at the Apical, Mid-ventricular and Basal levels was then visualized (Figure 3, panel C). Two independent observers estimated whether each segment contained more or less than 25% blood. The location of the inflow blood was determined in this way at two times during diastole: at mid-diastole, defined as halfway between peak flow during the E-wave and peak flow during the A-wave, and at end-diastole, defined as the time just before systolic contraction. The E-wave and A-wave were measured in the mitral annulus in the 4D PC-CMR flow data and used for timing reference only. Agreement between the observers was measured using the kappa coefficient [30].

## Results

### Volume Tracking

When using Volume Tracking, a starting volume of blood is first chosen. In Figure 2, Panel D the blood in the left atrium has been chosen, and the surface of this volume is displayed. As the blood flows, the volume will move and deform with the flow as if the volume's surface was infinitely flexible and stretchable, as seen in Figure 2, panel D-F, and Additional File 3: VT-Comparison.mpg. The Volume Tracking surface shows the boundary between inflowing blood and blood already in the ventricle from the previous heartbeat.

**Figure 4:**
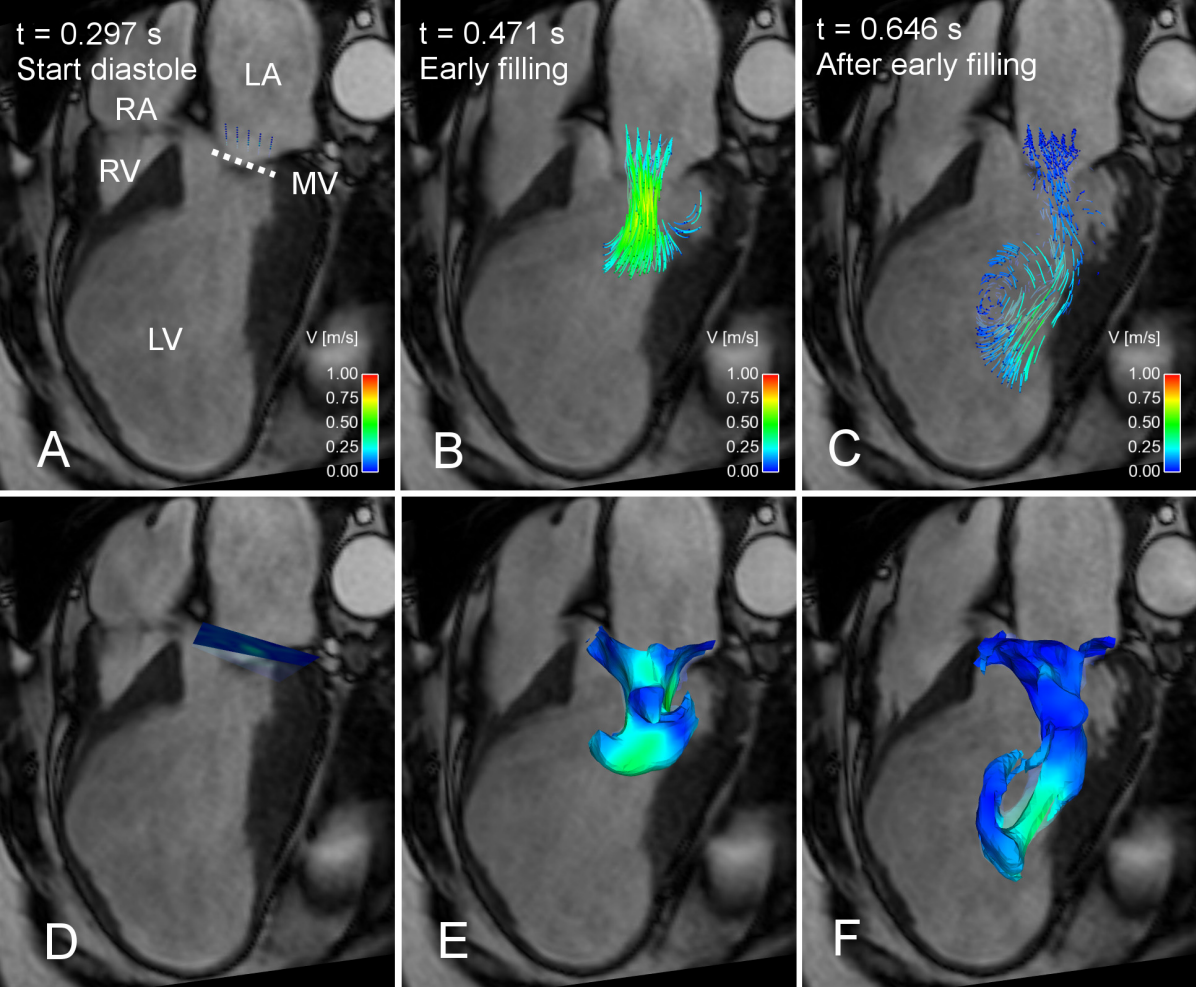
**Particle tracing and Volume Tracking visualizations of LV filling in a patient with apical aneurysm**. Particle tracing (A-C) and Volume Tracking (D-F) visualizations of LV filling in a patient with a large LV apical aneurysm, which appeared after a major anterior infarction. See Additional File 4: VT-Patient.mpg for an animated version. In each image, an anatomical 4-chamber Cine image is shown for orientation. The 4-chamber image is transparent to show flow behind the plane. Time is counted from the start of ventricular systole. In the particle trace images in panels A-C, particles are released every 20 milliseconds at the level of the white, dotted line (MV). In panel A, at the start of diastole, particles are shown at their starting position just above the dotted white line. In panel B, during early filling, the traces show the flow from the atrium into the ventricle. In panel C, at mid-diastole, the particles have moved further into the ventricle and entered a twisting pattern. Panels D-F show a Volume Tracking visualization corresponding to the particle trace visualization in panels A-C. In panel D, at the start of diastole, a Volume Tracking plane can be seen at the level of the mitral valve just before ventricular diastole. In panel E, during early filling, the plane has deformed and shows the blood flowing into the ventricle. In panel F, in mid-diastole, the blood has moved further into the ventricle. The inflow pattern is distinctly different from the pattern observed in the healthy volunteer in Figure 2. *LV = left ventricle, RV = right ventricle, LA = left atrium, RA = right atrium, MV = mitral valve, dotted line = approximate location of mitral valve, color = velocity from 0 (blue) to 1 m/s (red)*.

**Figure 5:**
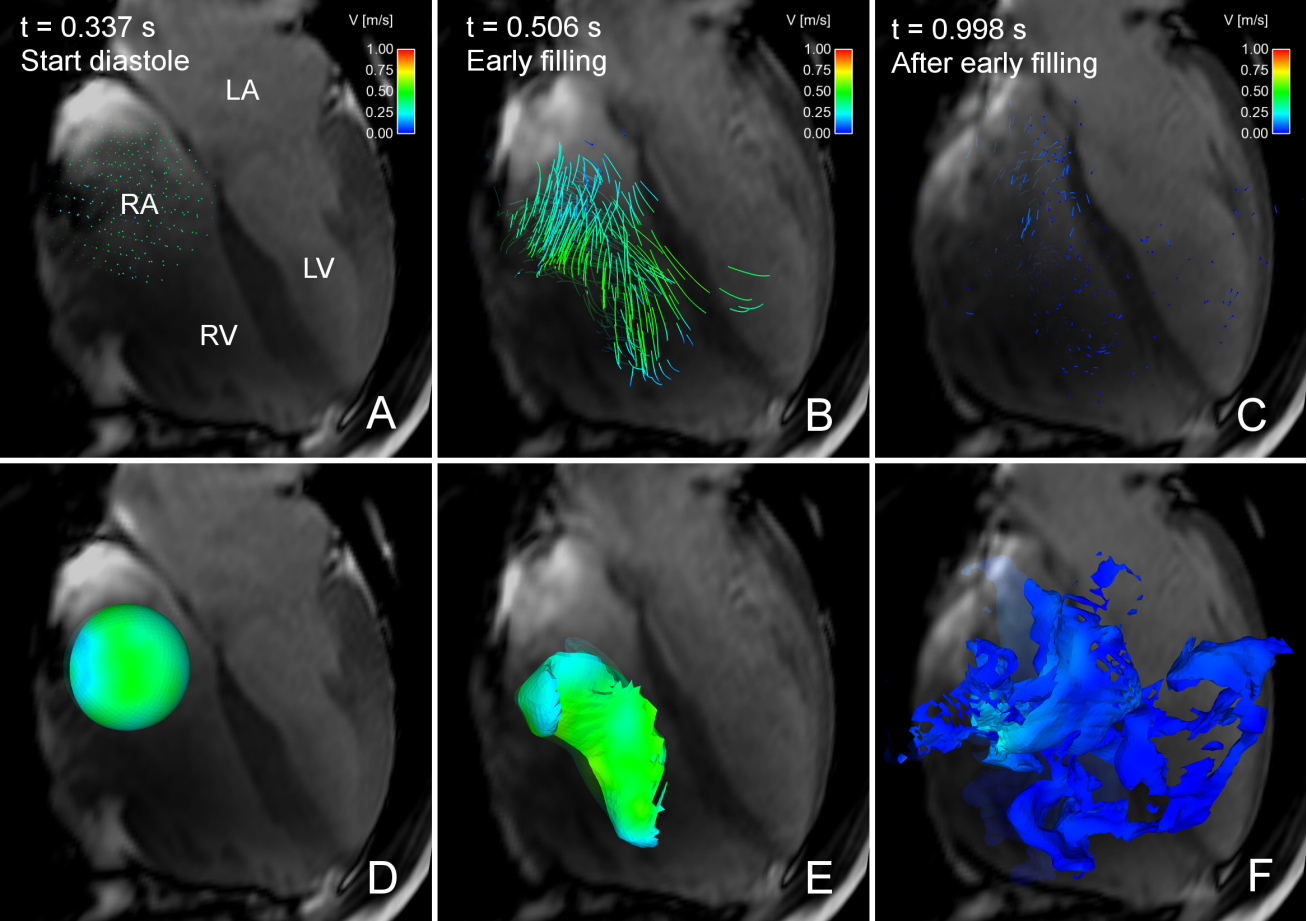
**Particle tracing and Volume Tracking visualizations of right ventricular filling flow**. Particle tracing (panels A-C) and Volume Tracking (panels D-F) visualizations of RV filling in volunteer 7. See Additional File 5: VT-RV.mpg for an animated version. In each image, an anatomical 4-chamber cine image is shown. The 4-chamber image is transparent to show flow behind the plane. The scene has been rotated in comparison to Figures 1, 2 and 4, to better show the visualizations. Due to the rotation, the RV is in the foreground, with the LV behind. Time is counted from the start of systole. Panels A-C show particle tracing. In panel A, at the start of diastole, a collection of particles has been placed in the right atrium. In panel B, during early filling, the particle traces move in a vortex pattern from the atrium into the ventricle. In panel C, at end-diastole, the particles have slowed down and spread in the right ventricle. Panels D-F show a Volume Tracking visualization corresponding to the particle tracing in panels A-C. Panel D, at the start of diastole, shows a spherical volume of blood in the right atrium. In Panel E, during early filling, the blood volume has deformed and flowed into the right ventricle. In Panel F, at end-diastole, the volume has been deformed further and has spread in the right ventricle. Comparing panels B and E, the vortex flow pattern is more apparent in the particle trace visualization in panel B. Comparing panels C and F, the Volume Tracking visualization in panel F shows how blood has spread in a complex pattern in the right ventricle. This is not apparent in the particle tracing visualization in panel C. *LV = left ventricle, RV = right ventricle, LA = left atrium, RA = right atrium, MV = mitral valve, color = velocity from 0 (blue) to 1 m/s (red)*.

Although only one shape is used in this example, a wide variety of sizes and shapes can be used. Planes are used in Figures 1, 2 and 4, and Figure 5 shows a sphere as initial volume. A more detailed discussion of the possible shapes can be found in Additional File 1: VT-Appendix.pdf. The formulation of the method allows the volume selection to be performed interactively using a point-and-click user interface.

### Evaluation and validation of Volume Tracking

In Figure 2 a comparison of visualizations of LV inflow using Volume Tracking and particle tracing is shown. Additional File 3: VT-Comparison.mpg shows an animated version of Figure 2, showing the effect of animation on Volume Tracking. Particle tracing shows an intricate pattern of swirling flows during diastolic filling, especially apparent in the animation (Additional File 3: VT-Comparison.mpg). Volume Tracking, in contrast, shows the boundary between the inflowing blood and the blood already in the ventricle. Additionally, Volume Tracking reveals an area close to the basal parts of the septum with no filling blood in subject 7 (Figure 2, lower right panel). This area was not observed when studying the particle trace visualization alone. Particle tracing shows a dynamic, complex scene, while Volume Tracking shows the combined movement of the whole inflow volume.

Figure 4 and Additional File 4: VT-Patient.mpg shows inflow into the left ventricle (LV) in a patient with a LV apical aneurysm, visualized using Volume Tracking and particle tracing. As in Figure 2, the visual impression of the LV inflow is different for the two methods. The inflow is distinctly different from the flow pattern in the healthy volunteer in Figure 2.

Figure 5 shows particle trace and Volume Tracking visualizations of inflow in the right ventricle (RV). Additional File 5: VT-RV.mpg shows an animated version of Figure 5. To demonstrate that Volume Tracking can be used with many different shapes, a sphere of blood in the right atrium (panel D) is used instead of a plane. The particle tracing visualization shows a vortex or helix motion of particles into the ventricle, and the particles coming to rest in the RV. Volume Tracking shows the initial volume deforming and moving into the right ventricle, but the vortex motion is not clearly visible. As the blood comes to rest in the RV during diastasis, it spreads out in a complex pattern. This complex pattern is not apparent in the particle tracing visualization. Additional File 5: VT-RV.mpg clearly shows the breakdown of ordered, vortical inflow to the complex deformation in late diastole.

Figure 1 shows the superposition of Volume Tracking and particle tracing visualizations of LV filling in a healthy volunteer. Additional File 2: VT-PT-Combination.mpg shows an animated version of Figure 1. Notice that very few particles pass through the plane. Table 2 shows the agreement between Volume Tracking and particle tracing, i.e. the fraction of particles that stay on the inflow side of the Volume Tracking surface. Mean agreement for all 9 subjects was 90.5% in mid-diastole and 87.8% in end-diastole. When varying the number of particles in subject 7 between 1000 and 8000, the standard deviation in the mid-diastolic agreement was 0.30% and in the end-diastolic agreement 0.17%.

**Table 2:** Validation.

	Subject	MD %	ED %
Normals:	1	90.0	88.4

	2	82.1	71.4

	3	96.4	95.5

	4	96.6	92.2

	5	90.3	88.8

	6	90.8	90.1

	7	90.6	89.7

	8	87.1	90.1

Patient:	AA	90.2	84.1

Mean ± SD:		90.5 ± 4.4	87.8 ± 6.8

Agreement between Volume Tracking and particle tracing, calculated as the percentage of released traces on the inflow side of the Volume Tracking surface (see Figure 1) at mid-diastole (MD) and end-diastole (ED). The mean agreement was 90.5% in mid-diastole and 87.8% in end-diastole.

### Analysis of LV inflow patterns

Semi-quantitative analysis of the inflow patterns was feasible in all subjects. The results of the flow pattern analysis are given in Tables 3 and 4 for Observer 1, and in Additional File 6: Distribution of LV inflow blood.xls. Agreement between the observers was excellent with a kappa coefficient of *κ *= 0.91. In seven out of eight volunteers an in the patient, blood did not reach the apical level by mid-diastole. In end-diastole, blood has reached the apical level in the majority of healthy subjects (observer 1: 6 of 8, observer 2: 8 of 8). In the patient, the inflowing blood did not reach the apical level at all. There was a large variability inflow patterns between the healthy volunteers. In the patient with ischemic cardiomyopathy, the inflow blood is only present in segments 4, 5, 6 and 7 in the basal third of the ventricle. This is notably different from the normals, where blood is present in several segments in the basal and mid-ventricular thirds.

**Table 3:** Distribution of left ventricular inflow blood for observer 1 at mid-diastole.

	Segment Apical							Mid-ventricular							Basal						
**Subject**	**1**	**2**	**3**	**4**	**5**	**6**	**7**	**1**	**2**	**3**	**4**	**5**	**6**	**7**	**1**	**2**	**3**	**4**	**5**	**6**	**7**

1								X	X	X	X	X	X	X	X	X	X	X	X	X	X

2								X	X	X	X	X			X			X	X		

3							X	X	X	X	X			X	X			X			

4								X	X		X	X		X					X	X	X

5								X	X	X	X			X		X	X	X	X	X	X

6									X	X		X	X	X	X	X	X	X	X	X	X

7								X	X		X	X		X	X		X	X	X		X

8								X	X			X	X	X	X	X	X	X	X	X	X

AA																			X		

This table shows the distribution of the blood flowing into the left ventricle in the volunteers (1-8) and the patient (AA) at mid-diastole for observer 1. Observer 1 at end-diastole can be seen in Table 4. The results from both observers can be seen in Additional file 6: Distribution of LV inflow blood.xls. Inflow blood locations were determined using Volume Tracking visualizations of LV filling (see Figure 2) and the segment model described in Figure 3. This table shows observer 1 at mid-diastole. Each row in the table shows one of the subjects included in the study. Columns correspond to segments in the segment model in Figure 3. Each segment was marked with an 'X' if the inflow blood occupied 25% or more of the area of the segment.

**Table 4:** Distribution of left ventricular inflow blood for observer 1 at end-diastole.

	Segment Apical							Mid-ventricular							Basal						
**Subject**	**1**	**2**	**3**	**4**	**5**	**6**	**7**	**1**	**2**	**3**	**4**	**5**	**6**	**7**	**1**	**2**	**3**	**4**	**5**	**6**	**7**

1						X	X	X	X	X			X		X	X	X	X	X	X	X

2							X				X	X	X	X	X			X	X	X	X

3								X	X	X				X			X	X	X		X

4	X								X	X	X	X		X	X		X	X	X	X	X

5							X	X	X	X	X			X	X	X	X	X	X	X	X

6	X						X	X	X	X	X	X		X	X	X	X	X	X	X	X

7							X	X		X	X	X		X	X		X	X	X	X	X

8								X	X		X	X	X	X	X	X	X	X	X	X	X

AA																		X	X	X	X

This table shows the distribution of the blood flowing into the left ventricle in the volunteers (1-8) and the patient (AA) at end-diastole for observer 1. Observer 1 at end-diastole can be seen in Table 4. The results from both observers can be seen in Additional file 6: Distribution of LV inflow blood.xls. Inflow blood locations were determined using Volume Tracking visualizations of LV filling (see Figure 2) and the segment model described in Figure 3. Each row in the table shows one of the subjects included in the study. Columns correspond to segments in the segment model in Figure 3. Each segment was marked with an 'X' if the inflow blood occupied 25% or more of the area of the segment.

## Discussion

In this article, a new method for visualization and analysis of 4D PC-CMR data, called Volume Tracking, is presented for the first time. Visually, Volume Tracking provides a different perspective on the flow data when compared to particle tracing. Volume Tracking and particle tracing have a mean agreement of 90.5% in mid-diastole and 87.8% in end-diastole. In the analysis of LV inflow patterns, observer agreement was excellent. The flow patterns varied between the normals, and in the ischemic cardiomyopathy patient, the inflow was notably different from the normals.

### Evaluation and validation of Volume Tracking

Three examples demonstrate that Volume Tracking provides incremental value to particle tracing: a) Volume Tracking shows the surface of the blood volume flowing into the left ventricle rather than the detailed dynamics; b) Volume Tracking emphasizes overall motion more than particle tracing; and c) Volume Tracking shows a complex flow pattern in RV filling not visible using particle tracing. This is in line with previous studies, where the visualization method influences the results of the visualization process [20,21]. However, this is the first study investigating the influence of the choice of visualization method for 4D PC-CMR data. The significance of this result may be that when visualizing and analyzing 4D PC-CMR data, researchers can extract more information from a given dataset using Volume Tracking. This may lead to new insights and additional knowledge about blood flow and cardiac pumping.

In general, it may be beneficial to use several complementary visualization methods when studying 4D PC-CMR data, since it may reveal more aspects of the flow. One example of this is that the vortex in the right ventricular inflow was less apparent with Volume Tracking than with particle tracing. To detect this type of flow patterns, vortex detection methods [14] and quantitative indices of helix flow [19,31] may be effective complements to Volume Tracking. Complementary to the large-scale flow dynamics shown by Volume Tracking, the flow dynamics in vessel wall boundary layers can be quantified using wall shear stress (WSS) [17] analysis. Simultaneous use of WSS analysis and Volume Tracking may provide a link between WSS and large-scale flow. Furthermore, the visual impression of the flow gained by Volume Tracking can be quantified by classifying particle traces according to origin and destination of flow [18,32].

The high agreement between Volume Tracking and particle tracing shows that Volume Tracking displays the same blood motion as the particle traces, which in this study were computed using a numerical algorithm independent from Volume Tracking and known to be accurate [28]. This suggests that the Volume Tracking numerical algorithm performs well in the presence of noise, acceleration and vortices in the 4D PC-CMR datasets used in this study. Additionally, the agreement between Volume Tracking and particle tracing shows that the differences in interpretation of the flow is not due to differences in blood transport calculations. The differences in interpretation of the flow may instead be explained by the differences in visual representation (surfaces instead of particles and lines). The low variation in agreement when changing the number of particles suggests that the agreement is not dependent on the number of particles.

In this study, only ventricular inflow has been studied. However, Volume Tracking is a general method, which may be used to study flow in all parts of the cardiovascular system. One example where Volume Tracking may also provide incremental value is the human aorta, where flow patterns may be influential in plaque formation and rupture.

### Analysis of LV inflow patterns

The physiological significance of the present findings may be an increased understanding of the complex, and clinically intangible, hemodynamics that may help clarify normal cardiac pumping. The different filling patterns in the healthy volunteers suggest that normal hemodynamics may display additional degrees of freedom that could explain differences in normal physiology.

The pathophysiological significance of the present findings may be that the transition from normal to pathological LV hemodynamics which may take several years to develop can be better understood and discovered at an earlier sub-clinical stage. These gradual changes over time may also be coupled to early morphological and physiological changes. In line with previous studies [33], we agree that flow patterns may be a sensitive measure of LV function and may be able to detect early stages of dysfunction.

### Limitations

Errors in the velocity measurements accumulate in particle paths and Volume Tracking surfaces over time [9]. This problem is minimized in this study by limiting the tracking to diastole.

Blood flow patterns may be influenced by factors which were not measured in the present experimental setting, such as changes in blood viscosity and hematocrit.

Long 4D PC-CMR acquisition times lead to averaging over several heartbeats, obscuring small flow details so that the resulting flow data describes large-scale flow features that are present in the majority of heartbeats. The 4D PC-CMR technique is also limited in spatial resolution, does not provide absolute pressure levels, and is not feasible during exercise. Noise in the 4D PC-CMR measurements and the complicated flow patterns in the human heart may also influence the Volume Tracking calcuations. The high agreement between particle tracing and Volume Tracking suggests that accuracy in the Volume Tracking algorithm is a minor problem for the present application.

Volume Tracking shows how a certain volume is deformed in the flow. This means that flow inside the volume is only seen indirectly. To overcome this limitation, Volume Tracking and particle tracing can be used simultaneously, as in Figure 1.

Ensight uses linear interpolation of the velocity fields in space and time to compute particle traces, which may introduce errors into the calculation. Since Ensight does not allow the user to choose interpolation method, the influence of the interpolation could not be checked. The authors are not aware of any studies of the effects of interpolation strategies in particle tracing, but the effects are probably small in relation to the smoothing, noise, low resolution and phase background effects of the 4D PC-CMR technique.

The evaluation of the visualization aspects of Volume Tracking was performed by visual comparison to particle tracing. An independent, quantitative evaluation for both methods would be desirable. However, the state of the art in evaluation methods for flow visualization has not yet been extended to treat time-resolved flow [20,21] and may not adress the hypothesis-generating aspects of visualization.

## Conclusion

In this study Volume Tracking, a new method for visualization of blood flow from 4D PC-CMR, is presented. Compared to particle tracing, Volume Tracking can provide incremental information that may lead to a better understanding of blood flow and may improve diagnosis and prognosis of cardiovascular diseases. The diastolic inflow pattern in the patient is notably different from the volunteers, suggesting that blood flow patterns may be used as a measure of LV function and as an early indicator of disease.

## Competing interests

The authors declare that they have no competing interests.

## Authors' contributions

JT developed the details of the method and implemented it in software, wrote the manuscript, created all figures and animations, performed analysis of LV inflow patterns and merged feedback from the other authors. MC collected data, supplied expertise in cardiac physiology and revised the manuscript for intellectual content. GS contributed to the design and development of the numerical method. HA supplied expertise in cardiac physiology and revised the manuscript for intellectual content. EH conceived the method, performed analysis of LV inflow patterns, contributed to the method design and revised the manuscript for intellectual content. All authors have read and approved the final manuscript.

## Pre-publication history

The pre-publication history for this paper can be accessed here:

http://www.biomedcentral.com/1471-2342/11/10/prepub

## Supplementary Material

Additional file 1 **Appendix: Theory and implementation**. Appendix: Document describing the mathematical theory and technical details of the implementation of Volume Tracking. Click here for file

Additional file 2 **Animation: Composite visualization of LV filling using both Volume Tracking and particle tracing**. Composite particle trace and Volume Tracking visualization of LV filling in volunteer 7, also shown in Figure 1. Anatomical four-chamber Cine images are displayed in the background for orientation. See Figure 1 for a description of the anatomy. The image is semi-transparent to show flow behind the four-chamber plane. Time is counted from the start of ventricular systole. At the beginning of the movie a Volume Tracking plane is visible at the level of the mitral valve, and a collection of particles are visible just above the plane. Particles are released every 20 milliseconds in the same location. The Volume Tracking plane can be seen as an infinitely flexible and stretchable sheet, deformed effortlessly with the flow. Between *t *= 0.337 s and *t *= 0.5 s, the Volume Tracking plane is deformed as blood flows into the ventricle. The particles show an ordered inflow. The Volume Tracking plane shows that the front of inflowing blood moves quickly towards the apex. After *t *= 0.5 s the particle traces show a complex arrangement of vortices, which slows down almost to a standstill around *t *= 0.8 s. The Volume Tracking surface keeps on deforming slowly even after *t *= 0.8s, when the particles seem to have stopped moving. Notice the correspondence between the two methods. Specifically, very few particles cross through the Volume Tracking plane. This is due to the fact that all particles are released in the inflowing blood and that the Volume Tracking plane separates the inflowing blood from the blood already in the ventricle. Click here for file

Additional file 3 **Animation: Comparison between particle tracing and Volume Tracking**. Visualization of LV filling in volunteer 7, also shown in Figure 2. Left: particle tracing. Right: Volume Tracking. The left and right panels differ only in the visualization method used. Anatomical four-chamber Cine images are displayed in the background for orientation. See Figure 2 for a description of the anatomy. The four-chamber plane image is semi-transparent to show flow behind the plane. The color scale indicates velocities between 0 and 1.0 meters per second. Time is counted from the start of ventricular systole. The video starts just before the opening of the mitral valve. In the left panel, particles are released every 20 milliseconds. In the right panel, a plane is placed near the mitral valve. The plane is infinitely flexible and stretchable and deforms effortlessly with the flow, showing the boundary between inflow blood and blood already in the ventricle. Between *t *= 0.337 s and t = 0.5 s, particle tracing shows an ordered inflow, and Volume Tracking shows the inflow blood progressing quickly into the ventricle. After *t *= 0.5 s, particle tracing shows a complex arrangement of vortices decelerating almost to standstill around *t *= 0.8 s. In contrast, Volume Tracking clearly shows a clockwise rotation and further deformation in the apical region after *t *= 0.8s. Additionally, Volume Tracking shows that inflow blood is located closer to the lateral wall of the LV in this subject. A region near the basal parts of the septum contains no filling blood (see Figure 2, white arrow), something which is not clearly visible using particle trace visualization. Click here for file

Additional file 4 **Animation: Particle trace and Volume Tracking visualizations of LV inflow in patient with apical aneurysm**. Particle tracing (left) and Volume Tracking (right) visualizations of LV inflow in a patient diagnosed with a large LV apical aneurysm. Anatomical four-chamber Cine images are displayed in the background for orientation. See Figure 4 for a description of the anatomy. The four-chamber image is transparent to show flow behind the four-chamber plane. The color scale in the lower left corner indicates velocities between zero and 1.0 meters per second (m/s). Time is counted from the start of ventricular systole. At the start of the movie, just before the filling of the LV begins, the starting location of the particles is visible just above the mitral valve (left). A Volume Tracking plane is visible at the level of the mitral valve (right). As the movie plays, the particles flow into the ventricle. The plane deforms to show the boundary between inflowing blood and blood already in the ventricle. Click here for file

Additional file 5 **Animation: Particle trace and Volume Tracking visualizations of right ventricular inflow**. Particle tracing (left) and Volume Tracking (right) visualizations of RV diastolic inflow. Anatomical four-chamber Cine images are displayed in the background for orientation. See Figure 5 for a description of the anatomy. The four-chamber image is transparent to show flow behind the four-chamber plane, and the scene has been rotated in comparison to Figures 1, 2 and 4. Due to the rotation, the RV is in the foreground, with the LV behind. The color scale in the lower left corner indicates velocities between zero and 1.0 meters per second (m/s). Time is counted from the start of ventricular systole. The animation starts at the beginning of RV filling. After this the animation can be divided into two parts. First an organized inflow into the right ventricle is shown between *t *= 0.337 s and *t *= 0.63 s. Particle tracing (left) shows a distinct swirling vortex of flow into the right ventricle. Volume Tracking does not show the vortex clearly. During the remaining time, from *t *= 0.63 s to *t *= 1.00 s, the flow decelerates during diastasis. Particle tracing shows the blood coming to rest in an uncomplicated manner. However, Volume Tracking shows the blood volume becoming stretched and twisted, finally occupying most of the right ventricle in a complex structure. The volume is spread out in three dimensions, outside the image plane, but is still completely within the RA and RV. Click here for file

Additional file 6 **Distribution of left ventricular inflow blood**. This file shows the distribution of left ventricular inflow blood as assessed by the two independent observers. Observer 1 is also shown in Tables 3 and 4. Click here for file
