# Supplementary material for: Volume Tracking: A new method for quantitative assessment and visualization of intracardiac blood flow from three-dimensional, time-resolved, three-component magnetic resonance velocity mapping
Source: BMC Med Imaging. 2011 Apr 12;11:10. doi: 10.1186/1471-2342-11-10 (PMC3102625; doi:10.1186/1471-2342-11-10)
Supplement: Additional file 1 — Appendix: Theory and implementation. Appendix: Document describing the mathematical theory and technical details of the implementation of Volume Tracking. [file 1471-2342-11-10-S1.PDF]

# Volume Tracking: A New Method for Visualization of Intracardiac Blood Flow from Three-Dimensional, Time-Resolved, Three-Component Magnetic Resonance Velocity Mapping
# Appendix: Theory and Numerical Implementation

Johannes Töger[1,2], Marcus Carlsson[1], Gustaf Söderlind[2], Håkan Arheden[1] and Einar Heiberg[1]

[1]Department of Clinical Physiology, Lund University, Skåne University Hospital Lund, Lund, Sweden
[2]Deparment of Numerical Analysis, Centre for Mathematical Sciences, Lund University, Lund, Sweden

April 1, 2011

## 1 Introduction

This document describes the theory and technical details of Volume Tracking, a new method for visualization of flow measured using four-dimensional phase contrast magnetic resonance imaging (4D PC-MRI) [1]. Volume Tracking provides a new visual impression of the flow compared to particle tracing, the most commonly used method for visualization of 4D PC-MRI data.

Volume Tracking uses a novel formulation of the flow tracking problem, where the motion of arbitrary volumes is encoded in a flow map. A partial differential equation (PDE) for the flow map is stated and solved using a finite volume scheme. This document describes the mathematical theory, PDE formulation and numerical solution, and is intended for readers interested in analyzing or reproducing the computational implementation of the method.

## 2 Theory and Notation

Let $\Omega \subset \mathbf{R}^3$ be the domain of interest, $\overline{x} = (x, y, z) \in \Omega$ the spatial coordinates, $t$ the physical time in some interval $I$ and $\overline{v}(\overline{x}, t)$ a velocity field defined on $\Omega \times I$.

To compute a particle's movement over time from a given velocity field, consider a particle moving through the velocity field, with a path described by the function $\overline{x}(t)$. Over a small time step $\Delta t$, the particle's movement is tangential to the local velocity, i.e.

$$\overline{x}(t + \Delta t) = \overline{x}(t) + \overline{v}(\overline{x}(t), t)\, \Delta t. \tag{1}$$

Rearranging this relation and taking the limit as $\Delta t \to 0$, i.e. taking smaller and smaller timesteps, gives the ordinary differential equation (ODE)

$$\frac{d}{dt}\overline{x}(t) = \overline{v}(\overline{x}(t), t) \tag{2}$$

for the particle's path through the flow. Equation 2 is the central assumption behind the particle trace technique [2], as well as for the method presented here.

Define the flow map $\phi_{t_0}^{t}(\overline{x})$ as the mapping of a particle's coordinates $\overline{x}$ at time $t_0$ to its coordinates at time $t$. A volume at a certain time $t_0$ whose evolution the user wants to study is called a *starting volume* and denoted $V_0$. The evolution of the volume is denoted $V(t)$, where $V(t_0) = V_0$. $V(t)$ can be seen as a set of points that is parameterized over time $t$. The evolution of the volume is defined as its image under the flow map:

$$V(t) = \phi_{t_0}^{t}(V_0). \tag{3}$$

## 3 Volume Tracking Theory

The novel feature of Volume Tracking is to represent a volume using a *volume function*. A starting volume $V_0$ is defined implicitly using the volume function $f : \Omega \mapsto \mathbf{R}$:

$$V_0 = \{\overline{x} \in \Omega \mid f(\overline{x}) \leq C\}, \tag{4}$$

i.e. at $t = t_0$ all particles fulfilling certain criteria on their coordinates are selected. Depending on $f$ and $C$, different shapes can be used, as summarized in Table 1. At another time, this representation cannot be used directly, as the particles will not be in the same position. However, using the flow map, particles can be selected by conditions on their coordinates at $t = t_0$:

$$V(t) = \left\{\overline{x} \in \Omega \mid f\left(\phi_{t}^{t_0}(\overline{x})\right) \leq C\right\}, \tag{5}$$

where $\phi$ is the flow map introduced above. Note that in this case, the flow map maps coordinates from the time $t$, where the volume is deformed, to time $t_0$, where the volume is in its initial state.

This observation is essential to the presented implementation of Volume Tracking. However, closed-form expressions for the flow map only exist for very simple flows. To compute the flow map for general velocity fields, Equation 2 could be used directly, but the computational cost would be prohibitive. Instead, consider that the particle coordinates at $t = t_0$ is a constant quantity along pathlines of the flow, as defined by Equation 2. In other words, the coordinates at $t = t_0$ are *advected* with the flow. Using the auxiliary function $\psi(\overline{x}, t) = \phi_t^{t_0}(\overline{x})$, and decomposing $\psi = (\psi^x, \psi^y, \psi^z)$ into its cartesian components, the advection equations

$$\begin{aligned}
\partial_t \psi^x + \overline{v} \cdot \nabla \psi^x = 0 \\
\partial_t \psi^y + \overline{v} \cdot \nabla \psi^y = 0 \\
\partial_t \psi^z + \overline{v} \cdot \nabla \psi^z = 0
\end{aligned} \quad (6)$$

hold in $\Omega$. Here $\nabla = (\partial_x, \partial_y, \partial_z)$ is the spatial gradient, $\partial_t$ is the partial derivative in time and the dot represents the dot product. The initial conditions at $t = t_0$ are straightforward:

$$\begin{aligned}
\psi^x(\overline{x}, t_0) = x \\
\psi^y(\overline{x}, t_0) = y \\
\psi^z(\overline{x}, t_0) = z.
\end{aligned} \quad (7)$$

Additionally, boundary conditions to Equation 6 are needed to ensure a unique solution, as particles that come into the domain must be assigned some origin. The most reasonable choice is to say that a particle coming from outside the domain originates at the boundary, i.e. to let

$$\begin{aligned}
\psi^x(\overline{x}, t) = x \\
\psi^y(\overline{x}, t) = y \\
\psi^z(\overline{x}, t) = z
\end{aligned} \quad (8)$$

at the parts of the boundary $\partial\Omega$ where inflow occurs.

# 4 Numerical implementation

Equation 6 consists of three non-coupled advection partial differential equations (PDEs). To obtain a solution in finite time, a numerical scheme must be used. In this study, a second-order finite volume scheme by LeVeque [3, 4] is used, as provided in the freely available software package CLAWPACK 4.3 [5]. The included advection module is used with full transverse propagation of increment and correction waves (method(3) = 22) and the monotonized centered (MC) limiter (mthlim = 4). The algorithm is second-order in space and time in

| $f$ | Shape | $C$ |
|---|---|---|
| $\|\overline{x}-\overline{a}\|_2$ | sphere at $\overline{a}$ | radius |
| $(\overline{x}-\overline{a})^T E(\overline{x}-\overline{a})$ | ellipsoid at $\overline{a}$ | size scale |
| $\overline{n}^T\overline{x}$ | half-space | position along $\overline{n}$ |
| $\|\overline{x}-\overline{a}\|_X$ (general norm) | convex set | size scale |

Table 1: Examples of the volume function $f$, the corresponding shape and interpretation of $C$. Volumes are represented as the set $\{\overline{x} \mid f(\overline{x}) \leq C\}$. Here $\overline{a}$ is an arbitrary point, and $E$ a symmetric positive definite matrix.

regions where the solution is smooth. Adaptive step-size control was turned on (method(1) = 1) with a target Courant-Friedrichs-Lewy (CFL) number of 0.95 (cflv(2) = 0.95).

When applying the numerical method to PC-MRI data, the domain was divided into cells identical to the measured voxels. The measured velocities represent voxel averages, but the numerical method described above requires the velocity evaluated at voxel interfaces. Therefore, the voxel interface velocity was taken as the average of the two adjacent voxel velocities. The measured velocities were supplied as variable coefficients in the advection solver.

Initial values are set to the appropriate coordinate component at the cell center before the computation starts. Boundary conditions are enforced by using two extra cells (ghost cells) around the boundary (mbc = 2). Their values are set to their center coordinate at each timestep.

All preprocessing of data was performed using Segment [6], a freely available software for cardiovascular image analysis, based on MATLAB (The Mathworks, USA). Ensight (CEI, USA) was used to compute particle traces for comparison, generate isosurfaces from the flow maps and generate animations.

# 5 Testing

To verify that the mathematical formulation, numerical method and implementation are correct and accurate, an artificial test case was constructed as follows. A three-dimensional swirling velocity field $\overline{v} = (u, v, w)$ in the domain $\Omega = [0, 1]^3$ defined by

$$\begin{aligned} u &= 2\sin^2(\pi x)\sin(2\pi y)\sin(2\pi z)\cos(\pi t/T), \\ v &= -\sin(2\pi x)\sin^2(\pi y)\sin(2\pi z)\cos(\pi t/T), \\ w &= -\sin(2\pi x)\sin(2\pi y)\sin^2(\pi z)\cos(\pi t/T) \end{aligned} \tag{9}$$

is considered, with $T = 1.5$ [3]. The field is divergence-free [3], i.e. models an incompressible flow, as can be expected for the present application. Due to the factor $\cos(\pi t/T)$ the flow reverses at $t = T/2$, so any volume tracked from $t = 0$ should deform and then return to its initial position at $t = T = 1.5$ in a

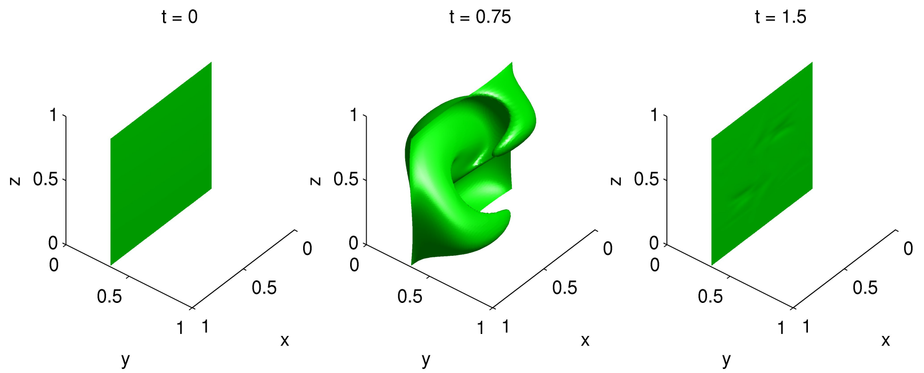


Figure 1: Test of the proposed implementation. The image shows a volume tracking visualization of a three-dimensional swirling flow, as defined in Equation 9, with T = 1.5. The expected result of the test is that the surface should deform and then return to its initial, flat shape at t = 1.5. Left: Initial surface, a plane at y = 0.35. Middle: The surface has been severely deformed. Right: The surface has returned to its starting position with a small error.

correct implementation. This test was chosen to capture the strong spatial and temporal variations of intracardiac blood flow, as well as to have a known exact solution for validation.

Figure 1 shows the performance of the proposed implementation using this test case. The resolution was 100 voxels (finite volume cells) in each direction. The velocity field in Equation 9 was evaluated at the midpoint of the voxel faces. It can be seen that the initial surface first deforms, and then returns to its starting position with a small error. The error in the flow map at $t = 1.5$, measured in $L_2(\Omega)$-norm, was 0.0012, or 0.12 voxels.

To test Volume Tracking with blood flow velocity data, the Volume Tracking solution was compared to particle traces, i.e. solutions to Equation 2. The particle traces were computed using a fourth-order Runge-Kutta ordinary differential equation (ODE) solver with adaptive timestepping, as implemented in the visualization software package Ensight (CEI, USA). The exact method and results of this comparison can be seen in the Volume Tracking article [1]. Particle tracing and Volume Tracking visualizations agreed well, with few particles passing through the surface of the Volume Tracking volume. This suggests that accuracy of the present implementation of Volume Tracking is comparable to the Runge-Kutta ODE solver for the purposes of the present visualization application.

# 6 Limitations and Future Work

The current implementation uses a constant spatial resolution, determined by the resolution of the acquired blood flow data. Additionally, the fixed spatial

resolution does not adapt to locally complex flow features such as vortices. Our experience is that the computed flow maps in real blood flow are smooth with localized sharp gradients. Accuracy in the solution may be determined by the resolution of these gradients. Therefore, a finite volume method with adaptive spatial resolution [7, 8] would be desirable.

The test case was chosen to be similar to real blood flow in that it is accelerating, fully three-dimensional and has a twisting effect. It also has a known solution for quantitative evaluation and is a standard test for advection solvers [3]. The test uses a smooth velocity field without the noise and blood/muscle boundaries present in 4D PC-MRI acquisitions, and the test may not hold for the more general situation. However, the comparison between Volume Tracking and particle tracing visualizations using real blood flow data, presented in the Volume Tracking article [1], Figure 3, shows that the Volume Tracking solution agrees well with the particle traces. Increased resolution and accuracy may be useful when studying more complex flow patterns, such as vortices and mixing flows.

# References


[1] Töger J, Carlsson M, Söderlind G, Arheden H, Heiberg E: **Volume Tracking: A New Method for Quantification and Visualization of Intracardiac Blood Flow from Three-Dimensional, Time-Resolved, Three-Component Magnetic Resonance Velocity Mapping**. *BMC Medical Imaging* 2011. [In Press].

[2] Wigström L, Ebbers T, Fyrenius A, Karlsson M, Engvall J, Wranne B, Bolger A: **Particle Trace Visualization of Intracardiac Flow Using Time-Resolved 3D Phase Contrast MRI**. *Magnetic Resonance in Medicine* 1999, **41**(4):793–799.

[3] LeVeque R: **High-Resolution Conservative Algorithms for Advection in Incompressible Flow**. *SIAM Journal on Numerical Analysis* 1996, **33**(2):627–665.

[4] Langseth J, LeVeque R: **A Wave Propagation Method for Three-Dimensional Hyperbolic Conservation Laws**. *Journal of Computational Physics* 2000, **165**(1):126–166.

[5] LeVeque R, Berger M: **CLAWPACK Software 4.3** [`http://www.clawpack.org/`]. [Accessed 2009-10-23].

[6] Heiberg E, Sjögren J, Ugander M, Carlsson M, Engblom H, Arheden H: **Design and validation of Segment- freely available software for cardiovascular image analysis**. *BMC Medical Imaging* 2010, **10**:1.

[7] Berger M, Oliger J: **Adaptive mesh refinement for hyperbolic partial differential equations**. *Journal of computational Physics* 1984, **53**(3):484–512.

[8] Bell J, Berger M, Saltzman J, Welcome M: **Three-dimensional adaptive mesh refinement for hyperbolic conservation laws**. *SIAM Journal on Scientific Computing* 1994, **15**:127.
